# Supplementary material for: Associations between Parental Stress and Subsequent Changes in Dietary Intake and Quality among Preschool Children Susceptible to Obesity
Source: Int J Environ Res Public Health. 2021 Mar 30;18(7):3590. doi: 10.3390/ijerph18073590 (PMC8038074; doi:10.3390/ijerph18073590)
Supplement: Supplementary file 1 [file ijerph-18-03590-s001.zip › Supplementary table S1.docx]

**Supplementary Material**

**Table S1: Parental Stress Index**

| Questions selected and modified from the Swedish version of the Parental Stress Index Questions on parental stress: *“Which changes have you experienced in your life after you had your child/children?”* | |
| --- | --- |
| □ Less sleep  □ More sleep  □ No change | □ Less work  □ More work  □ No change |
| □ Less stress  □ More stress  □ No change | □ Fewer social gatherings in the home  □ More social gatherings in the home  □ No change |
| □ Fewer worries  □ More worries  □ No change | □ Less joy of life  □ More joy of life  □ No change |
| □ Less time to yourself  □ More time to yourself  □ No change | □ Less everyday surplus energy  □ More everyday surplus energy  □ No change |
| □ Fewer household conflicts  □ More household conflicts  □ No change | □ Less complexity of being a parent compared to expectations  □ More complexity of being a parent compared to expectations  □ As expected |
| Other__________________________________ | |
