# Supplementary material for: Associations between Parental Stress and Subsequent Changes in Dietary Intake and Quality among Preschool Children Susceptible to Obesity
Source: Int J Environ Res Public Health. 2021 Mar 30;18(7):3590. doi: 10.3390/ijerph18073590 (PMC8038074; doi:10.3390/ijerph18073590)
Supplement: Supplementary file 1 [file ijerph-18-03590-s001.zip › Supplementary table S2.docx]

**Supplementary Material**

**Table S2. Definition of food groups.**

| **Fruit:** fresh, canned and frozen (excluding jam, fruit juice, dried fruit and fruit products with added sugar). |
| --- |
| **Vegetables:** fresh, canned and frozen (excluding fried onion, ketchup, pickles and potatoes) |
| **Fish:** fatty fish, lean fish and shellfish. |
| **Sugar sweetened beverages:** soft drinks, squash, chocolate milk, milkshake and drinking yoghurt. |
| **Starch:** rice, pasta and potatoes. |
