# Supplementary material for: Associations between Parental Stress and Subsequent Changes in Dietary Intake and Quality among Preschool Children Susceptible to Obesity
Source: Int J Environ Res Public Health. 2021 Mar 30;18(7):3590. doi: 10.3390/ijerph18073590 (PMC8038074; doi:10.3390/ijerph18073590)
Supplement: Supplementary file 1 [file ijerph-18-03590-s001.zip › Supplementary table S3.docx]

**Supplementary Material**

|  |  | **Parental Stress** |  |
| --- | --- | --- | --- |
|  | **N** | **β (95% CI) ^*^** | **P** |
| **Total energy (KJ/day)** |  |  |  |
| Crude | 495 | –6.05 (–47.24; 35.13) | 0.77 |
| Adjusted ^1^ | 495 | –10.55 (–51.89; 30.80) | 0.62 |
| **Protein (E%)** |  |  |  |
| Crude | 495 | –0.02 (–0.14; 0.11) | 0.81 |
| Adjusted ^1^ | 495 | –0.02 (–0.14; 0.11) | 0.81 |
| **Carbohydrates (E%)** |  |  |  |
| Crude | 495 | 0.03 (–0.18; 0.23) | 0.80 |
| Adjusted ^1^ | 495 | 0.02 (–0.19; 0.23) | 0.85 |
| **Added sugar (E%)** |  |  |  |
| Crude | 495 | –0.09 (–0.72; 0.55) | 0.78 |
| Adjusted^1^ | 495 | –0.01 (–0.68; 0.66) | 0.98 |
| **Fat (E%)** |  |  |  |
| Crude | 495 | –0.01 (–0.25; 0.22) | 0.92 |
| Adjusted ^1^ | 495 | 0.01 (–0.24; 0.26) | 0.93 |
| **Saturated fat (E%)** |  |  |  |
| Crude | 495 | –0.05 (–0.26; 0.16) | 0.61 |
| Adjusted ^1^ | 495 | –0.07 (–0.28; 0.13) | 0.47 |
| **Fruit (g/day)** |  |  |  |
| Crude | 495 | 0.44 (–1.67; 2.58) | 0.68 |
| Adjusted ^1^ | 495 | –0.06 (–2.19; 2.07) | 0.96 |
| **Vegetables (g/day)** |  |  |  |
| Crude | 495 | –0.85 (–4.02; 2.32) | 0.59 |
| Adjusted ^1^ | 495 | –1.30 (–4.53; 1.93) | 0.42 |
| **Fish (g/day)** |  |  |  |
| Crude | 495 | 0.46 (–0.51; 1.43) | 0.35 |
| Adjusted ^1^ | 495 | 0.39 (–0.58; 1.35) | 0.42 |
| **Starch (g/day)** |  |  |  |
| Crude | 495 | –0.57 (–2.11; 0.97) | 0.46 |
| Adjusted ^1^ | 495 | 0.64 (–2.17; 0.88) | 0.40 |
| **Sugar sweetened beverages (g/day)** |  |  |  |
| Crude | 495 | 0.25 (–3.72; 4.23) | 0.90 |
| Adjusted ^1^ | 495 | 0.49 (–3.69; 4.67) | 0.81 |
| **DQI (units) ^§^** |  |  |  |
| Crude | 495 | 0.01 (–0.02; 0.04) | 0.52 |
| Adjusted^1^ | 495 | 0.00 (–0.02; 0.03) | 0.90 |
| ^*^All analysis is adjusted for baseline measure of outcome. ^1^: Adjusted for (intervention/control), age, sex, BMI z–score, sleep, physical activity, and maternal BMI education, and physical activity. **^§^:** Diet quality index | | | |

**Table S3. Association between parental stress at baseline and subsequent change in child dietary intake and quality index (multiple imputations).**
