# Supplementary material for: Associations between Parental Stress and Subsequent Changes in Dietary Intake and Quality among Preschool Children Susceptible to Obesity
Source: Int J Environ Res Public Health. 2021 Mar 30;18(7):3590. doi: 10.3390/ijerph18073590 (PMC8038074; doi:10.3390/ijerph18073590)
Supplement: Supplementary file 1 [file ijerph-18-03590-s001.zip › Supplementary material 2_informed consent control group-2.doc]

Projekt Sund Start

Enheden for Epidemiologisk Kostforskning

Institut for Sygdomsforebyggelse

Øster Søgade 18

1357 København K

___________________________________________

Erklæring om Samtykke.

_________________________________________________________________

Institut for Sygdomsforebyggelse gennemfører for tiden et interventionsprojekt. Projektet Sund Start er et forskningsprojekt, som har til formål at øge vores viden om, hvordan vi kan forebygge udvikling af overvægt blandt de 2-5 årige børn, som er i særlig risiko for at udvikle overvægt og fedme.

I forbindelsen med projektet tilbydes familierne en vejledning i sunde vaner. Derudover måles på barnets fedmegrad og deres kostvaner.

Vi anmoder hermed forældre/barnets værge om tilladelse til, at bruge data i forskningsøjemed. Alle oplysninger der indsamles i forbindelse med projektet forbliver strengt fortrolige. Vi har tavshedspligt, og ingen oplysninger om enkelte personer gives videre. I forskningsøjemed behandles alle deltagerne derudover under fuld anonymitet. Deltagelse i interventionen er frivillig, familierne kan til enhver tid trække sig ud af projektet.

Jeg/vi bekræfter hermed, at jeg/vi efter at have modtaget overstående information, indvilliger i, at vores/mit barn deltager i det beskrevne projekt. Jeg/vi er informeret om, at det er frivilligt at deltage, og at tilsagnet når som helst kan trækkes tilbage.

Jeg giver samtykke til, at____________________________________________________________

(Barnets navn og CPR-nummer)

deltager i interventionsprojektet Sund Start og har læst ovenstående skriftlige information om projektet ________(Sæt kryds)

Vi ønsker ikke at deltage, men projektet må beholde allerede indsamlede data ________ (sæt kryds)

Vi ønsker ikke at deltage i projektet _______(Sæt kryds)

Særlige forhold/bemærkninger _______________________________________________________

Navnet eller navnene på forældremyndighedens indehaver(e):

________________________________________________________________________________

Dato: ______ Underskrift: __________________________________________________________
